# Characteristics of Silicone Oil Emulsification After Vitrectomy for Rhegmatogenous Retinal Detachment: An Ultrasound Biomicroscopy Study

**DOI:** 10.3389/fmed.2021.794786

**Published:** 2022-01-13

**Authors:** Hongmei Zhao, Jian Yu, Yuan Zong, Chunhui Jiang, Haohao Zhu, Gezhi Xu

**Affiliations:** ^1^Department of Ophthalmology and Vision Science, Eye and Ear, Nose and Throat Hospital, Fudan University, Shanghai, China; ^2^Key Laboratory of Myopia of State Health Ministry, Shanghai, China; ^3^Key Laboratory of Visual Impairment and Restoration of Shanghai, Shanghai, China; ^4^National Health Commission (NHC) Key Laboratory of Myopia, Fudan University, Shanghai, China; ^5^Key Laboratory of Myopia, Chinese Academy of Medical Sciences, Shanghai, China; ^6^Department of Ophthalmology, Shanghai Fifth People's Hospital, Fudan University, Shanghai, China

**Keywords:** silicone oil, emulsification, ultrasound biomicroscopy, vitrectomy, rhegmatogenous retinal detachment

## Abstract

**Purpose:** To investigate the characteristics of silicone oil (SO) emulsification after vitrectomy for rhegmatogenous retinal detachment (RRD) and possible correlations with clinical factors.

**Methods:** Patients who underwent primary pars plana vitrectomy with SO injection for RRD followed by SO removal at the Eye and ENT Hospital of Fudan University between January 2016 and January 2020 were included. Ultrasound biomicroscopy (UBM) images of the anterior segment were taken before SO removal. Eight signs of SO emulsification in the UBM images were graded as 1 (present) or 0 (not present) and the grades for all signs in each eye were summed. Correlations between SO emulsification grade and clinical factors were determined.

**Results:** A total of 118 patients (118 eyes) were enrolled in this study. Emulsified SO particles were found in all 118 eyes (100%). The eight signs were more frequently detected in the superior part of the eye. The mean total SO emulsification grade was 19.99 ± 12.98 (range: 1–36). Younger age and male (both *P* < 0.05) were associated with higher total SO emulsification grade. Patients with intraocular pressure (IOP) > 21 mmHg or the use of antiglaucoma medications at the time of SO removal had a higher total SO emulsification grade, were younger, and were more frequently male (all *P* < 0.05) than patients without ocular hypertension.

**Conclusions:** UBM could play an important role in the diagnosis and grading of SO emulsification. Younger patients and males are more prone to SO emulsification, which may lead to elevated IOP.

## Introduction

Silicone oil (SO), first introduced by Cibis et al. in the 1960s (1), is now widely used in the management of complicated retinal detachment. However, complications of this procedure have been reported, the most frequent of which are SO emulsification and glaucoma (2, 3). The incidence of glaucoma following SO tamponade was reported to range from 11 to 56% (4, 5), exceeding the frequency in cases without SO tamponade (6). SO emulsification was proposed as a major reason for this complication (3). Many methods have been used to detect and evaluate SO emulsification, including ultrasound biomicroscopy (UBM). UBM can be used to take high-resolution images of the anterior segment and has improved our understanding of many ocular diseases, especially glaucoma. Azzolini et al. (7) and Grigera et al. (8) described the UBM findings of SO emulsification in the anterior segment. However, those studies did not fully explore the correlations between UBM findings and other clinical characteristics, such as glaucoma. Therefore, we used UBM to study SO emulsification in the anterior segment in a group of patients who underwent vitrectomy and SO tamponade for rhegmatogenous retinal detachment. We also explored the potential correlations between UBM and the clinical findings.

## Materials and Methods

### Study Subjects and Ethics Statement

This was a single-center, observational, cross-sectional study. Patients who underwent primary pars plana vitrectomy (PPV) with SO injection for rhegmatogenous retinal detachment (RRD), followed by SO removal, at the Eye and ENT Hospital of Fudan University between January 2016 and January 2020 were enrolled in the study. The study was approved by the Institutional Review Board of the Eye and ENT Hospital of Fudan University, and conformed to the tenets of the Declaration of Helsinki. All of the patients signed an informed consent form.

Standard three-port, 23-gauge PPV was performed in all patients by a single surgeon (CH Jiang) using the Alcon Constellation system (Alcon Laboratories, Inc., Fort Worth, TX, USA). After central vitreous removal, triamcinolone acetonide was injected to visualize the residual posterior hyaloid, which was then removed. Additional procedures such as membrane peeling, perfluorocarbon liquid injection (DK-line), relaxing retinotomy, and inferior peripheral iridectomy were performed depending on the state of the retina. After fluid–air exchange and endophotocoagulation, SO (5700 cSt; Bausch & Lomb Inc., Rochester, NY, USA) was injected.

Patients with a history of trauma, SO injection, or intraocular surgery other than PPV or cataract surgery, an intraocular disease other than RRD or cataract (e.g., glaucoma, uveitis), elevated intraocular pressure (IOP > 21 mmHg) before PPV, diabetes, or age <18 years at the time of primary PPV were excluded from the study.

### Main Ophthalmic Measurements

Before SO removal, each patient underwent a thorough ophthalmic examination, which included assessment of best-corrected visual acuity [BCVA, logarithm of the minimal angle of resolution (logMAR)], calculation of spherical equivalent power (calculated as one-half of the cylindrical dioptric plus the spherical diopter), slit-lamp microscopy, dilated fundus examination with a non-contact lens (Maxfield 84 Diopter; Ocular, USA), measurement of IOP by non-contact tonometry, measurement of axial length (AL) using an IOLmaster (version 3.01; Carl Zeiss Meditec, Jena, Germany), and UBM (MD-300L, 50-MHz probe transducer; Meda Co., Ltd, Tianjin, China). We also collected demographic and clinical histories, including the presence of choroidal detachment, whether or not combined phacoemulsification was performed during PPV, history of intraocular operation, duration of SO *in situ*, lens status (aphakic, pseudophakic, or phakic), and the usage and number of antiglaucoma medications before SO removal.

### Examination Methods

UBM was performed by experienced ophthalmologists using the protocol described by Grigera et al. (8) and Avitabile et al. (9) UBM was performed with the patients lying in a supine position. The gain was set at 81 dB. Tetracaine 1% was applied for topical anesthesia. An eye cup with a diameter of 18–24 mm was placed into the conjunctival sac according to the size of the eye, and was then filled with care solution or normal saline. A central scan in the horizontal axis and additional scans in eight clock directions (12:00, 1:30, 3:00, 4:30, 6:00, 7:30, 9:00, and 10:30) were taken.

### Image Analysis Methods

**Figure 1:**
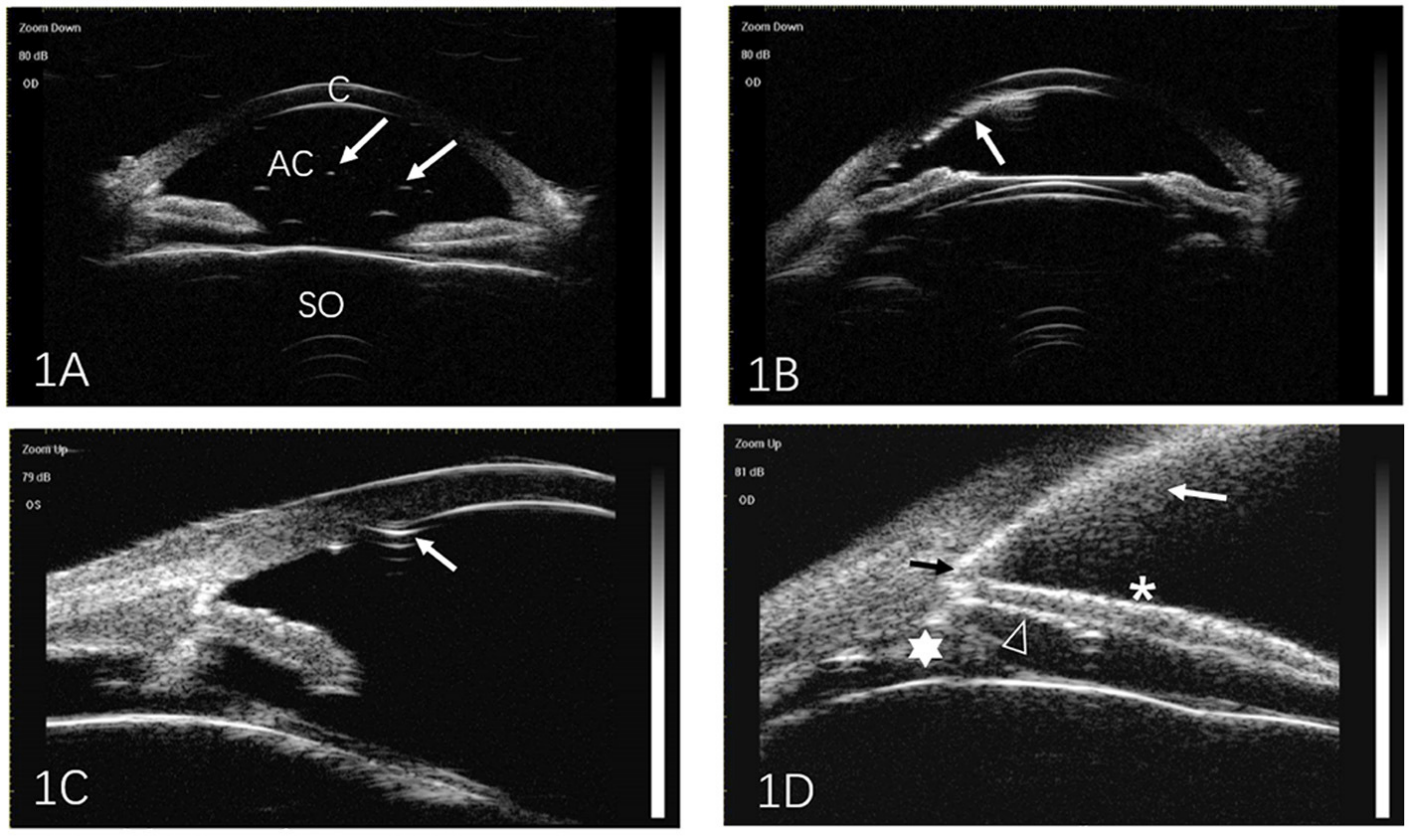
Representative ultrasound biomicroscopic images of silicone oil (SO) emulsification. **(A)** Individual SO particles floating in the anterior chamber (arrows). **(B)** SO particles adjacent to the cornea (arrow). **(C)** Ghost images. Multiple echoes from SO particles adjacent to the corneal endothelium may be seen as needle-shaped artifacts hanging from the endothelium (arrow). **(D)** Hyperoleon. A massive collection of emulsified SO particles (white arrow), and impregnation of the anterior chamber angle (black arrow), anterior surface of the iris (asterisk), posterior surface of the iris (triangle), and ciliary body (star). C, cornea; AC, anterior chamber; SO, silicone oil.

All UBM images were analyzed by two independent readers. The extent of SO emulsification detected by UBM was classified according to the method described by Grigera et al. (8). The following five signs were considered to indicate SO emulsification. (1) Floating droplets: tiny reflective particles (droplets) with definite outlines, floating in AC (Figure 1A). (2) Endothelial deposits with a fixed status (Figure 1B). (3) Ghost images (Figure 1C): multiple echoes of SO particles adjacent to the corneal endothelium that may be visible as needle-shaped images hanging from the endothelium. (4) Hyperoleon: a massive collection of emulsified particles (Figure 1D). (5) Impregnation or impregnation of tissues (Figure 1D): increased reflectivity in the anterior chamber angle (ACA) filtration area, the anterior/posterior iris surface, or the ciliary body, as compared with that in normal eyes.

**Figure 2:**
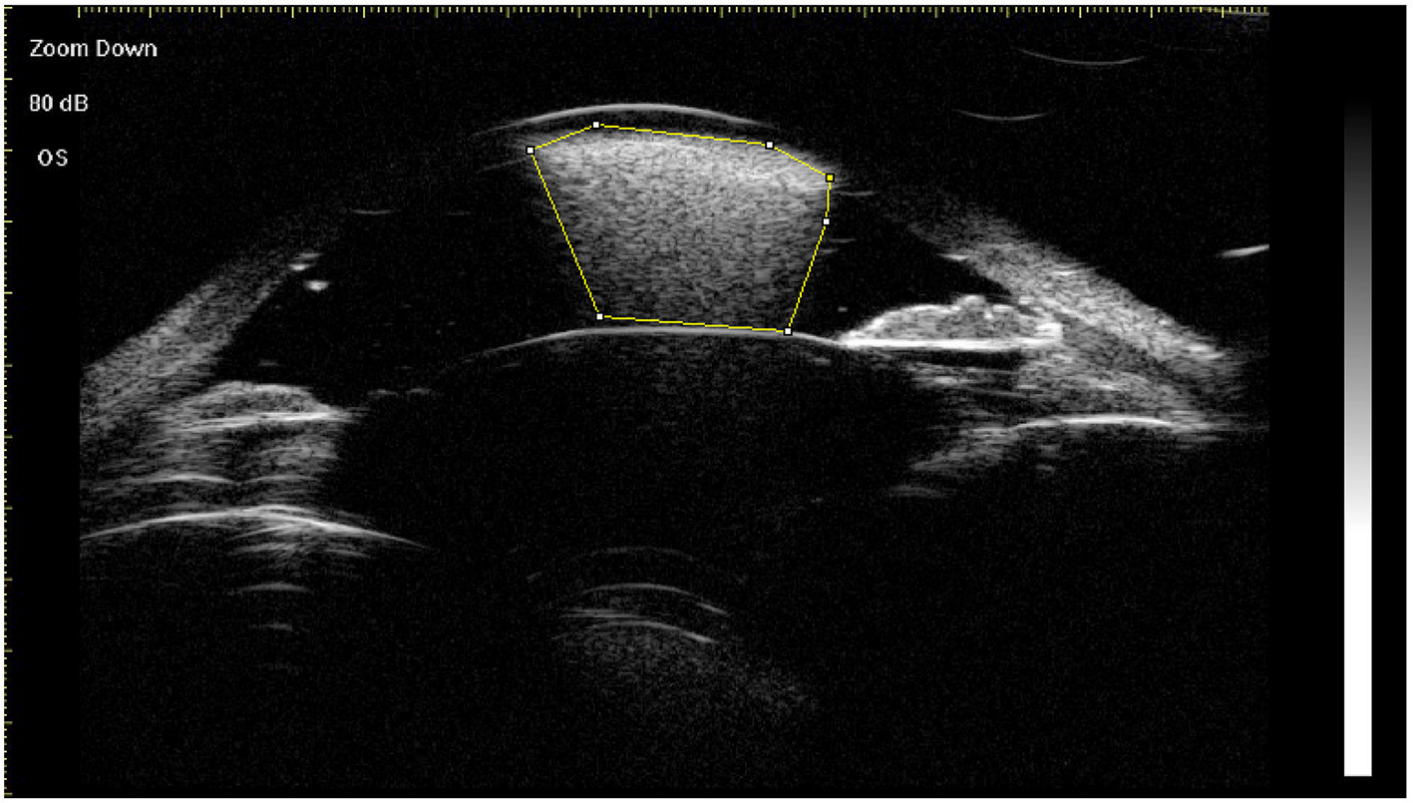
Measurement of the area of hyperoleon (indicated with the yellow border) in the anterior chamber using ImageJ software.

Using the horizontal central scan image, floating droplets, endothelial deposits with a fixed status, ghost images, and hyperoleon were graded as 1 (present) or 0 (not present) (8). The area of hyperoleon was measured using ImageJ software (National Institute of Health, Bethesda, MD, USA) (Figure 2). Impregnation of tissue at four locations (ACA, anterior iris surface, posterior iris surface, and ciliary body) was graded as 1 (present) or 0 (not present) on the images acquired in the eight clock directions, as reported by Grigera et al. (8). If the grades determined by both graders were identical, they were used as the final grades. If the grades differed, the final grade was determined by a senior specialist (Qian Chen). The grades for all signs in each eye were summed (Supplementary Figure 1). Intraobserver repeatability and interobserver reproducibility were evaluated for 20 central scan images by two observers, who each measured the same scan from each eye twice. The intraclass correlation (ICC) coefficient was used to assess repeatability and reproducibility. The mean of three measurements of the area of hyperoleon was used as the final value.

### Data and Statistical Analysis

All analyses were performed using SPSS software version 20.0 (SPSS, Inc., Chicago, IL, USA). The K–S test was used to determine the normality of data. The χ^2^ test, independent-samples *t*-test, Spearman's correlation coefficient, Mann–Whitney U test and multiple linear regression were used to assess correlations between clinical characteristics and SO emulsification. The independent sample *t*-test, χ^2^ test or Mann–Whitney U test were used to assess the differences between patients with/without elevated IOP (IOP > 21 mmHg) or the use of antiglaucoma medications. Continuous data are expressed as the mean ± standard deviation. Statistical significance was defined as a *P* value of < 0.05. ICC coefficients were used to assess repeatability and reproducibility; an ICC coefficient of 0.81–1.00 indicates almost perfect agreement between repeated measurements and values <0.40 indicate poor to fair agreement.

## Results

A total of 118 eyes (69 right) in 118 patients (59 males) were included in this study. The mean age of the sample was 55.76 ± 10.76 years (range 24–78 years), the mean duration of SO *in situ* was 23.72 ± 14.53 weeks (range 2–96 weeks), and the mean AL was 26.68 ± 2.92 mm (range 22–37 mm). Thirteen patients had choroidal detachment at the time of PPV. Fifty-two eyes were highly myopic (AL ≥ 26 mm). Combined phacoemulsification, without intraocular lens implantation, was performed in 51 eyes (Supplementary Table 1).

**Figure 3:**
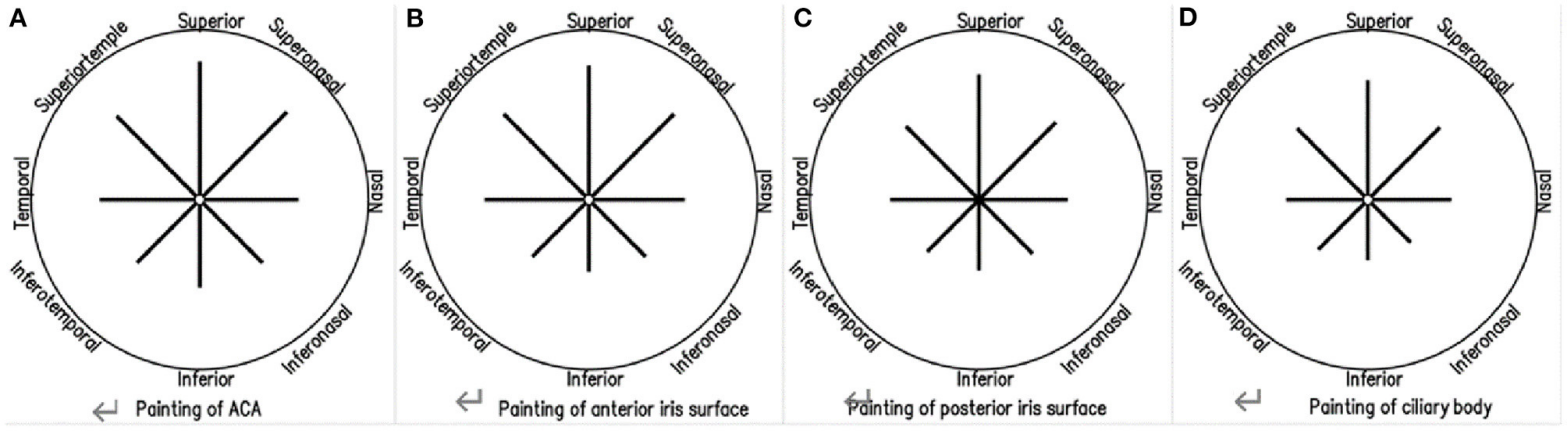
Ultrasound biomicrosopic findings of emulsified silicone oil in eight directions, showing impregnation of the anterior chamber angle **(A)**, the anterior iris surface **(B)**, the posterior iris surface **(C)**, and the ciliary body **(D)**.

Before SO removal, signs of SO emulsification were detected in all 118 eyes (100%). Floating droplets were found in 100%, hyperoleon in 20.33%, endothelial deposits in 28.81%, ghost images in 37.29%, impregnation of the ACA in 83.90%, impregnation of the anterior iris surface in 81.36%, impregnation of the posterior iris surface in 79.66%, and impregnation of the ciliary body in 77.97% of eyes. The mean ICC coefficient for measurement of the area of hyperoleon was 0.998 for intra-observer repeatability and 0.997 for interobserver reproducibility. When different directions were involved, tissue impregnation was most frequently detected in the superior part (Supplementary Table 2; Figure 3).

The mean total SO emulsification grade was 19.99 ± 12.98 (range:1–36). Age (negatively), duration of SO *in situ* (positively), AL (positively), combined phacoemulsification (positively), and male (positively) were significantly correlated with the total SO emulsification grade (all *P* < 0.05; Table 1). Multiple linear regression revealed that younger age and male (both *P* < 0.05) were associated with higher total SO emulsification grade (Table 2).

**Table 1:** Associations between clinical characteristics and total SO emulsification grade.

**Characteristic**	**Total grade/correlation coefficient**	***U* value**	***P* value**
Gender: female/male	15.80 ± 12.85 vs. 24.194 ± 11.79	1094.5	0.001*
Combined phacoemulsification with PPV+SO injection: yes/no	25.73 ± 11.05 vs. 18.37 ± 13.08	773.55	0.006^*^
Choroidal detachment: yes/no	22.23 ± 13.93 vs. 19.71 ± 12.90	583.0	0.392
Lens status: phakic/non-phakic	27.67 ± 9.77 vs. 18.61 ± 13.04	508.5	0.003^*^
Age	*r* = −0.313	-	0.001^*^
AL	*r* = 0.230	-	0.012^*^
BCVA before PPV + SO injection (logMAR)	*r* = 0.153	-	0.098
Duration of SO *in situ*	*r* = 0.192	-	0.037^*^

* *P < 0.05 was considered statistically significant. Mann–Whitney U test or Spearman's correlation coefficients were used to assess correlations between clinical characteristics and total SO emulsification grade.*

*SO, silicone oil; PPV, pars plana vitrectomy; AL, axial length; BCVA, best-corrected visual acuity; logMAR, logarithm of the minimum angle of resolution*.

**Table 2:** Results of multiple linear regression of factors associated with the total SO emulsification grade.

**Clinical factor**	**β coefficient (95%CI)**	***P* value**
Gender: female/male	11.173 (5.142, 17.203)	0.000^*^
Age	−0.364 (0.669, −0.060)	0.019^*^
Duration of SO *in situ*	0.164 (−0.047, 0.375)	0.127
AL	0.902 (0.181, 1.984)	0.102
Combined phacoemulsification with PPV + SO injection: yes/no	−5.207 (−13.622, 3.208)	0.223
Lens status: non-phakic/phakic	3.899 (−4.465, 16.2279)	5.881
Choroidal detachment: yes/no	1.924 (−8.287 12.135)	0.710
BCVA before PPV + SOI (logMAR)	1.417 (−0.766, 3.601)	0.201

* *P < 0.05 was considered statistically significant.*

*SO, silicone oil; AL, axial length; pars plana vitrectomy; BCVA, best-corrected visual acuity; logMAR, logarithm of the minimum angle of resolution*.

At the time of SO removal, 69 patients had an IOP of > 21 mmHg or were using antiglaucoma medications. These patients had a higher SO emulsification grade, a larger area of hyperoleon, were younger, and were more frequently males than patients without ocular hypertension (all *P* < 0.05) (Table 3).

**Table 3:** Comparison of clinical factors between patients with and without ocular hypertension (IOP > 21 mmHg or use of antiglaucoma medications).

**Clinical factor**	**Ocular hypertension**		***P* value**
	**Yes (*n* = 69)**	**No (*n* = 49)**	
Total grade	22.70 ± 12.64	16.18 ± 12.62	0.005^*^
Duration of SO *in situ* (weeks)	24.30 ± 15.71	22.90 ± 12.80	0.778
Age (years)	52.83 ± 10.11	59.91 ± 10.36	0.000^*^
AL (mm)	27.03 ± 2.94	26.18 ± 2.86	0.105
BCVA before PPV + SO injection (logMAR)	2.17 ± 1.06	2.08 ± 1.00	0.450
Male/female (n)	40/29	19/30	0.040* χ^2^ = 4.223
Choroidal detachment, yes/no (n)	60/9	45/4	0.404 *χ^2^* = 0.696
Combined phacoemulsification with PPV + SO injection: yes/no (n)	51/18	41/8	0.207 *χ^2^* = 1.589
Lens status: non-phakic/phakic (n)	14/55	4/45	0.071 *χ^2^* = 3.259

* *P < 0.05 was considered statistically significant.*

*Independent-samples t-tests, χ^2^ tests and Mann–Whitney U tests were used to compare clinical factors between patients with without ocular hypertension (IOP > 21 mmHg or the use of antiglaucoma medications). Values are expressed as the number of patients or as the mean ± standard deviation.*

*IOP, intraocular pressure; SO, silicone oil; AL, axial length; BCVA, best-corrected visual acuity; log MAR, logarithm of the minimal angle of resolution; PPV, pars plana vitrectomy*.

## Discussion

In this study, we used UBM to assess the presence of and to grade SO emulsification in a group of patients with RRD. SO emulsification was more commonly found in the superior direction, and was more severe in younger patients and in males. Furthermore, the SO emulsification grade was higher in patients with IOP > 21 mmHg or patients using antiglaucoma medications.

Several studies have observed SO emulsification using UBM. Avitabile et al. (9) determined the height of SO emulsification in the AC, and Grigera et al. (8) described various signs of SO emulsification. However, the correlations between signs of SO emulsification on UBM and clinical factors have not been well-studied. In our study, signs of SO emulsification in different directions were recorded by UBM and analyzed semi-quantitatively, and we explored their potential correlations with clinical factors (Supplementary Table 3). We found that the grade of SO emulsification was correlated with younger age and male gender. Previous studies (4, 10) also reported a correlation between SO emulsification and age. Aqueous proteins and eye movement, which facilitate SO emulsification and are greater in younger eyes, might be contributing factors (11). Greater activity may have contributed to the higher grade of SO emulsification in males in our study. Patients who underwent combined phacoemulsification during PPV possibly experienced more severe postoperative reactions or inflammation, which could contribute to their higher grade of SO emulsification. In addition, AL was significantly and positively correlated with the grade of SO emulsification. The reason for this is not fully understood, but it is possible that eyes with a longer AL were injected with a greater volume of SO, thus increasing the interface between SO and intraocular fluid. With a greater interface, the surfactants (surface active agents) have more opportunity to interact with SO, thus increasing the risk of emulsification (4). These factors might contribute to the higher grading observed in this study. In our study, UBM focused on the anterior segment. Weakened zonular laxity in highly myopic eyes (12) might permit emulsified SO droplets to enter the posterior and anterior chambers more easily.

Our study found the correlation between SO emulsification and elevated IOP, which was also observed in prior studies (9, 13). Wickham et al. used transmission electron microscopy and detected SO in the trabecular meshwork (14). In addition, Rentsch et al. (15) reported SO-laden macrophages within the trabecular meshwork of three human eyes, which were enucleated due to glaucoma following SO injection. It has also been proposed that SO might initiate localized inflammation in the trabecular meshwork (14, 16). Formerly Federman and Schubert (2) reported that 100% of eyes injected with SO showed some degree of emulsification. And this time, SO emulsification was observed in every eye included in our study. Here, we found that impregnation of the ACA was more frequent in patients with IOP > 21 mmHg or using antiglaucoma medications (Supplementary Table 4). As a result, SO emulsification seemed to be almost inevitable, also as it is closely associated with high IOP or the usage of antiglaucoma medications, so in these cases, emulsified SO droplets should be removed as thorough as possible. On the other hand, our result here reminds us that though presently most attention was paid to removing the SO droplets in the vitreous cavity, the anterior segment should also be taken care. Anterior chamber irrigation should be performed during silicone oi removal, especially in cases with high IOP.

Here, some of our findings were in accordance with former reports, this accordance suggested the UBM method used here a useful and reliable tool to evaluate the situation of SO emulsification. Compared to other ways like a Coulter Counter (10, 17, 18) and B-scan ultrasonography (19–21), UBM was non-invasive and cost effective, furthermore it could be used before SO removal, while the other two only postoperatively. On the other hand, slit-lamp microscopy, which could also be used before SO removal, was rather subjective and difficult to be quantified. Using the method described here, the situation of SO emulsification could be properly evaluated, and for those with higher sore, special attention should be paid to the removal of the SO droplets, and longer irrigation and multiple air-fluid exchange should be performed (18). Other methods including the recently introduced F4H5 (19), which is believed to dissolve the emulsified SO droplets, could also be considered.

Our study was limited by its single-center, cross-sectional design and we only included patients with RRD who were treated with one type of SO. On the other hand, we followed the standard described by Grigera DE, these are possibility that signs like impregnation of tissues could also result from reasons other than emulsified SO droplets.

In conclusion, UBM might play an important role in the diagnosis and quantification of SO emulsification. Younger patients and males may be more prone to SO emulsification and at increased risk of elevated IOP. SO should be removed as soon as possible in these patients. The method used to evaluate SO emulsification could be used in future studies in this field.

## Data Availability Statement

The original contributions presented in the study are included in the article/Supplementary Material, further inquiries can be directed to the corresponding authors.

## Ethics Statement

The studies involving human participants were reviewed and approved by the Institutional Review Board of the Eye and ENT Hospital of Fudan University. The patients/participants provided their written informed consent to participate in this study.

## Author Contributions

CJ performed the surgery. HZha and JY analyzed the patient data and made major contributions for writing the manuscript. YZ performed the literature review for similar topics and made major contributions to acquisition and interpretation of data. HZhu and GX made substantial contributions to conception and design this study. All authors have read and approved the final manuscript.

## Funding

Publication of this article was supported, in part, by research grants from the National Key Research and Development Plan (2017YFC0108200), the Shanghai Committee of Science and Technology (19441900900 and 201409006800), and National Natural Science Foundation of China (82070980).

## Conflict of Interest

The authors declare that the research was conducted in the absence of any commercial or financial relationships that could be construed as a potential conflict of interest.

## Publisher's Note

All claims expressed in this article are solely those of the authors and do not necessarily represent those of their affiliated organizations, or those of the publisher, the editors and the reviewers. Any product that may be evaluated in this article, or claim that may be made by its manufacturer, is not guaranteed or endorsed by the publisher.

## Supplementary Material

Supplementary Figure 1 Ultrasound biomicroscopic images from a single eye.
