# Supplementary material for: Characteristics of Silicone Oil Emulsification After Vitrectomy for Rhegmatogenous Retinal Detachment: An Ultrasound Biomicroscopy Study
Source: Front Med (Lausanne). 2022 Jan 13;8:794786. doi: 10.3389/fmed.2021.794786 (PMC8793062; doi:10.3389/fmed.2021.794786)
Supplement: Supplementary file 1 [file Table_1.DOCX]

**Supplementary Table 1.** Patient demographics and clinical characteristics

| **Variable** | **Value** |
| --- | --- |
| Number | 118 |
| Sex (male/female) | 59/59 |
| Age (years) | 55.76 ± 10.76 (24–78) |
| Left/right eye | 49/69 |
| Choroidal detachment (yes/no) | 13/105 |
| High myopia (yes/no) | 52/66 |
| AL (mm) | 26.68 ± 2.92 (22.00–37.00) |
| IOP (mmHg) | 17.63 ± 5.74 (6.0–37.0) |
| Mean duration of SO in situ (weeks) | 23.72± 14.53 (2–96) |
| Lens state (aphakic/phakic/pseudophakic) | 76/18/24 |
| Use of antiglaucoma medications (yes/no) | 65/53 |

*Values are expressed as the number of patients or as the mean ± standard deviation (range).*

*AL, axial length; IOP, intraocular pressure; SO, silicone oil*
