# Supplementary material for: Characteristics of Silicone Oil Emulsification After Vitrectomy for Rhegmatogenous Retinal Detachment: An Ultrasound Biomicroscopy Study
Source: Front Med (Lausanne). 2022 Jan 13;8:794786. doi: 10.3389/fmed.2021.794786 (PMC8793062; doi:10.3389/fmed.2021.794786)
Supplement: Supplementary file 2 [file Table_2.DOCX]

**Supplementary Table 2.** Frequencies of patients with signs of SO emulsification and grade in eight directions

| **UBM sign** | **N (%)** | **Grade on eight directions** |
| --- | --- | --- |
| Impregnation of the ACA | 110 (83.97%) | 5.02 ± 3.27 |
| Impregnation of the anterior iris surface | 106 (80.92%) | 4.83 ± 3.21 |
| Impregnation of the posterior iris surface | 104 (79.39%) | 4.42 ± 3.27 |
| Impregnation of the ciliary body | 81 (63.78%) | 4.20 ± 3.22 |

*UBM, ultrasound biomicroscopy; ACA, anterior chamber angles.*
