# Supplementary material for: Characteristics of Silicone Oil Emulsification After Vitrectomy for Rhegmatogenous Retinal Detachment: An Ultrasound Biomicroscopy Study
Source: Front Med (Lausanne). 2022 Jan 13;8:794786. doi: 10.3389/fmed.2021.794786 (PMC8793062; doi:10.3389/fmed.2021.794786)
Supplement: Supplementary file 3 [file Table_3.DOCX]

Supplementary Table 3. Associations between clinical characteristics and UBM findings

| **UBM findings** | **Clinical factor** | | | | | | |
| --- | --- | --- | --- | --- | --- | --- | --- |
|  | **Gender** | **Combined phacoemulsification** | **Choroidal detachment** | **Aphakic lens** | **Age** | **AL** | **Duration of SO in situ** |
| Hyperoleon | χ2 = 3.348  P = 0.109 | χ2 = 9.933  P = 0.004* | χ2 = 0.981  P = 0.532 | χ2 = 7.617  P = 0.015* | r = −0.183  P = 0.048* | r = 0.097  P = 0.292 | r = 0.115  P = 0.214 |
| Ghost images | χ2 = 3.624  P = 0.057 | χ2 = 5.937  P = 0.027* | χ2 = 0.491  P = 0.692 | χ2 = 5.155  P = 0.045* | r = −0.232  P = 0.011* | r = 0.135  P = 0.145 | r = 0.099  P = 0.285 |
| Endothelial deposits | χ2 = 5.950  P = 0.025* | χ2 = 4.889  P = 0.449 | χ2 = 0.027  P = 0.869 | χ2 = 4.648  P = 0.061 | r = −0.252  P = 0.006* | r = 0.127  P = 0.170 | r = 0.144  P = 0.121 |
| Impregnation of the ACA | t = −3.537  P = 0.001* | t = 3.115  P = 0.003* | t = −0.733  P = 0.463 | t = 2.512  P = 0.002* | r = −0.269  P = 0.003* | r = 0.279  P = 0.002* | r = 0.230  P = 0.012* |
| Impregnation of the anterior iris surface | t = −3.534  P = 0.001* | t = 2.001  P = 0.048* | t = −0.842  P = 0.401 | t = −3.756  P = 0.001* | r = −0.350  P = 0.000* | r = 0.224  P = 0.015* | r = 0.169  P = 0.067 |
| Impregnation of the posterior iris surface | t = −3.137  P = 0.002* | t = 0.163  P = 0.048* | t = −0.403  P = 0.637 | t = −2.052  P = 0.042* | r = −0.282  P = 0.002* | r = 0.244  P = 0.008* | r = 0.209  P = 0.023* |
| Impregnation of the ciliary body | t = −4.056  P = 0.000* | t = 0.592  P = 0.013* | t = −0.338  P = 0.736 | t = −2.737  P = 0. 007* | r = −0.296  P = 0.001* | r = 0.181  P = 0.050 | r = 0.164  P = 0.075 |

**P < 0.05 was considered statistically signiﬁcant. Independent-samples t tests, χ2 tests, and Pearson’s correlation coefficient were used to assess correlations between clinical characteristics and UBM findings.*

*AL, axial length; SO, silicone oil; ACA, anterior chamber angle.*
