# Supplementary material for: Characteristics of Silicone Oil Emulsification After Vitrectomy for Rhegmatogenous Retinal Detachment: An Ultrasound Biomicroscopy Study
Source: Front Med (Lausanne). 2022 Jan 13;8:794786. doi: 10.3389/fmed.2021.794786 (PMC8793062; doi:10.3389/fmed.2021.794786)
Supplement: Supplementary file 4 [file Table_4.DOCX]

Supplementary Table 4. Comparison of signs of SO emulsification between patients with or without ocular hypertension (IOP > 21 mmHg or use of antiglaucoma medications)

| **Factor** | **Ocular hypertension** | | **P Value** |
| --- | --- | --- | --- |
|  | **Yes (n = 69)** | **No (n = 49)** |  |
| Tissue impregnation, grade | 14.57 ± 12.01 | 20.65 ± 11.88 | 0.007* |
| Impregnation of the ACA, grade | 5.58 ± 3.14 | 4.16 ± 3.25 | 0.019* |
| Impregnation of the anterior iris surface, grade | 3.82 ± 3.09 | 5.43 ± 3.07 | 0.006* |
| Impregnation of the posterior iris surface, grade | 3.29 ± 3.15 | 5.10 ± 3.14 | 0.002* |
| Impregnation of the ciliary body, grade | 3.31 ± 3.11 | 4.67 ± 3.14 | 0.022* |
| Ghost image, grade | 1.71 ± 1.56 | 3.09 ± 2.62 | 0.001* |
| Hyperoleon, grade | 0.82 ± 1.75 | 1.71 ± 2.52 | 0.025* |
| Floating droplets, grade | 6.73 ± 2.26 | 7.38 ± 1.62 | 0.093 |
| Endothelial deposits, grade | 1.96 ± 2.97 | 2.70 ± 3.25 | 0.204 |
| Area of hyperoleon (mm2) | 66.26 ± 14.27 | 17.95 ± 94.4 | 0.029* |

**P < 0.05 was considered statistically signiﬁcant. Independent-samples t tests were used to assess differences between patients with or without ocular hypertension (IOP > 21 mmHg or use of antiglaucoma medications).*
