# Supplementary figures and images for: Characteristics of Silicone Oil Emulsification After Vitrectomy for Rhegmatogenous Retinal Detachment: An Ultrasound Biomicroscopy Study
Source: Front Med (Lausanne). 2022 Jan 13;8:794786. doi: 10.3389/fmed.2021.794786 (PMC8793062; doi:10.3389/fmed.2021.794786)

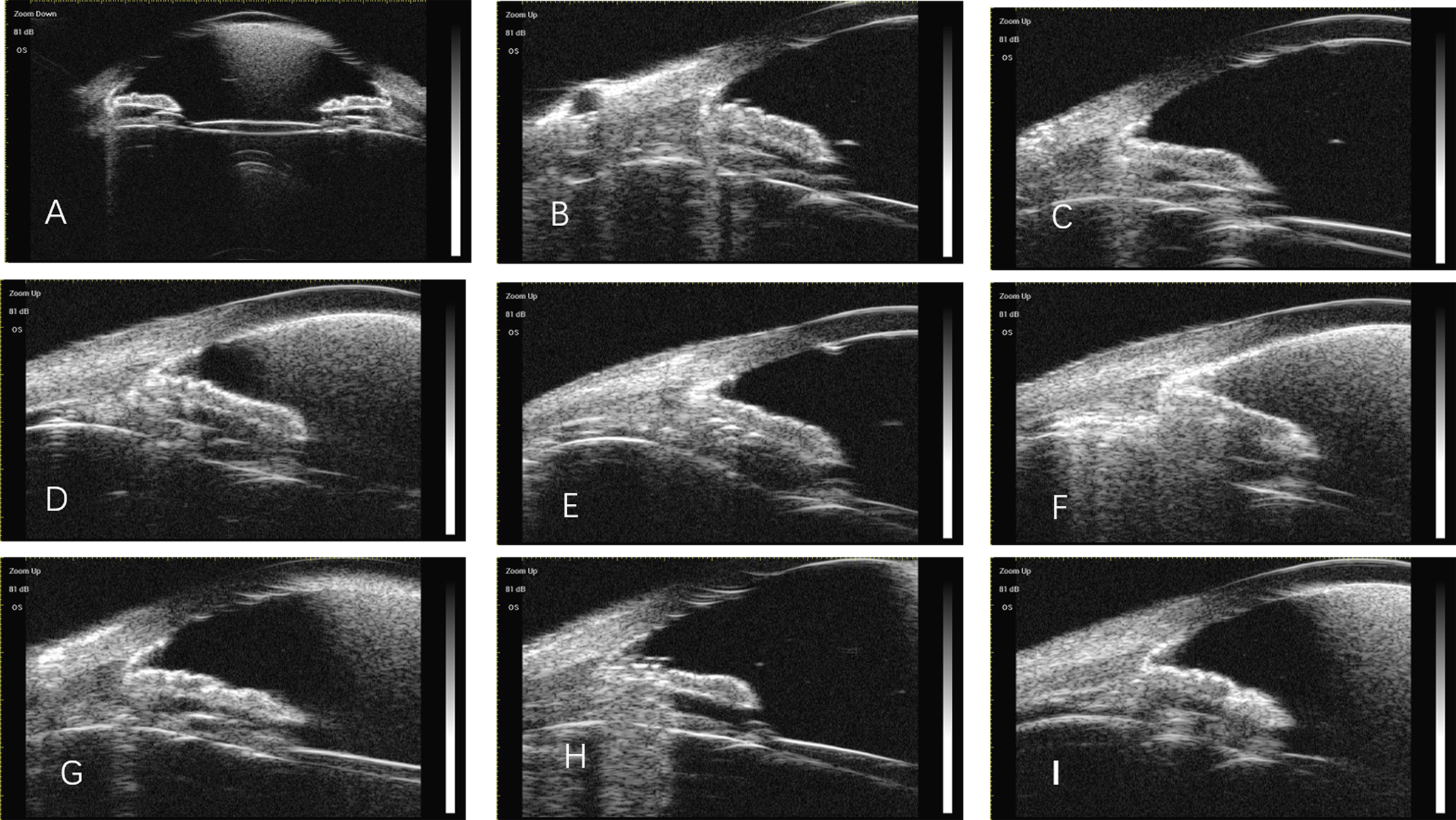

Supplement: Supplementary Figure 1 — Ultrasound biomicroscopic images from a single eye. [file Image_1.TIF]
